# Explainable machine learning for materials discovery: predicting the potentially formable Nd–Fe–B crystal structures and extracting the structure–stability relationship

**DOI:** 10.1107/S2052252520010088

**Published:** 2020-09-23

**Authors:** Tien-Lam Pham, Duong-Nguyen Nguyen, Minh-Quyet Ha, Hiori Kino, Takashi Miyake, Hieu-Chi Dam

**Affiliations:** a Japan Advanced Institute of Science and Technology, 1-1 Asahidai, Nomi, Ishikawa 923-1292, Japan; b ESICMM, National Institute for Materials Science, 1-2-1 Sengen, Tsukuba, Ibaraki 305-0047, Japan; c Center for Materials Research by Information Integration, National Institute for Materials Science, 1-2-1 Sengen, Tsukuba, Ibaraki 305-0047, Japan; d CD-FMat,AIST, 1-1-1 Umezono, Tsukuba, Ibaraki 305-8568, Japan; e JST, PRESTO, 4-1-8 Honcho, Kawaguchi, Saitama 332-0012, Japan

**Keywords:** data mining, machine learning, materials informatics, first-principles calculations, new magnets

## Abstract

New Nd–Fe–B crystal structures can be formed via the elemental substitution of *LA*–*T*–*X* host structures, including lanthanides (*LA*), transition metals (*T*) and light elements, *X* = B, C, N and O. The 5967 samples of ternary *LA*–*T*–*X* materials that are collected are then used as the host structures. For each host crystal structure, a substituted crystal structure is created by substituting all lanthanide sites with Nd, all transition metal sites with Fe and all light-element sites with B. High-throughput first-principles calculations are applied to evaluate the phase stability of the newly created crystal structures, and 20 of them are found to be potentially formable. A data-driven approach based on supervised and unsupervised learning techniques is applied to estimate the stability and analyze the structure–stability relationship of the newly created Nd–Fe–B crystal structures. For predicting the stability for the newly created Nd–Fe–B structures, three supervised learning models: kernel ridge regression, logistic classification and decision tree model, are learned from the *LA*–*T*–*X* host crystal structures; the models achieved maximum accuracy and recall scores of 70.4 and 68.7%, respectively. On the other hand, our proposed unsupervised learning model based on the integration of descriptor-relevance analysis and a Gaussian mixture model achieved an accuracy and recall score of 72.9 and 82.1%, respectively, which are significantly better than those of the supervised models. While capturing and interpreting the structure–stability relationship of the Nd–Fe–B crystal structures, the unsupervised learning model indicates that the average atomic coordination number and coordination number of the Fe sites are the most important factors in determining the phase stability of the new substituted Nd–Fe–B crystal structures.

## Abstract 2

Twenty new Nd–Fe–B crystal structures can be found via the elemental substitution of 5967 host structures of lanthanides–transition metals–light elements collected from the Open Quantum Materials Database. The average atomic coordination number and coordination number of the Fe sites are the most important factors in determining the structure–stability relationship of the new substituted Nd–Fe–B crystal structures.

## 1. Introduction   

The major challenge in finding new stable material structures in nature requires high-throughput screening of an enormous number of candidate structures, which are generated from different atomic arrangements in three-dimensional space. In fact, only a handful of structures among these candidates are likely to exist. Therefore, for the non-serendipitous discovery of new materials, candidate structures must be generated strategically so that the screening space is reduced without overlooking potential materials.

Multiple strategies have been proposed for the high-throughput screening processes (Butler *et al.*, 2018 ▸; Curtarolo *et al.*, 2013 ▸; Saal *et al.*, 2013 ▸) for finding various new materials. Almost all well known screening methods consider first-principles calculations as the basis for the estimation of physical properties. Screening processes have been successfully developed for theoretically understanding rare-earth-lean intermetallic magnetic compounds (Körner *et al.*, 2016 ▸, 2018 ▸), Heusler compounds (Ma *et al.*, 2017 ▸; He *et al.*, 2018 ▸; Balluff *et al.*, 2017 ▸), topological insulators (Yang *et al.*, 2012 ▸; Li *et al.*, 2018 ▸), perovskite materials (Emery *et al.*, 2016 ▸; Michalsky & Steinfeld, 2017 ▸), cathode coatings for Li-ion batteries (Aykol *et al.*, 2016 ▸) and *M*_2_ *AX* compounds (Ashton *et al.*, 2016 ▸). In recent years, various screening processes have been used to replace canonical approaches by machine learning (ML) methods. A few notable works based on ML models involve searching for hard-magnetic phases (Möller *et al.*, 2018 ▸), Heusler compounds (Kim *et al.*, 2018 ▸), bimetallic facet catalysts (Ulissi *et al.*, 2017 ▸), BaTiO3-based piezoelectrics (Xue *et al.*, 2016*b* ▸), polymer dielectrics (Mannodi-Kanakkithodi *et al.*, 2016 ▸), perovskite halides (Pilania *et al.*, 2016 ▸) and low-thermal-hysteresis NiTi-based shape memory alloys (Xue *et al.*, 2016*a* ▸).

ML is expected to play three different roles in performing screening processes. The first role is to replace the density functional theory (DFT) calculation and reduce the calculation cost of physical property estimation, *e.g.* convex hull distance (Kim *et al.*, 2018 ▸) and adsorption energy (Ulissi *et al.*, 2017 ▸). The reported models have achieved reasonable results in statistical evaluation tests such as cross validation. However, ensuring the reliability of extrapolating the physical properties of new materials is a major problem because the new screening materials do not always possess the same distribution as the training materials.

The second role of ML is to increase the success rate in screening processes. Given a list of hypothetical structures, ML methods are utilized for recommending the most likely new potential materials using probabilistic models [*e.g.* Bayesian optimization techniques (Yamashita *et al.*, 2018 ▸; Xue *et al.*, 2016*b* ▸)]. This approach requires a list of potential candidates to be prepared as input, which is primarily based on human intuition. The bottleneck of the current recommendation methods is that a large number of known property materials are required as references for the system to start an effective recommendation process. This number increases dramatically with the material description dimension. Furthermore, the computational cost of the recommended process increases significantly with the number of reference materials.

The third role of ML is to effectively generate new structure candidates. The notable algorithms for this purpose are random search-based algorithms (Pickard & Needs, 2006 ▸, 2007 ▸, 2011 ▸; Wang *et al.*, 2010 ▸; Zhang *et al.*, 2017 ▸), evolutionary-algorithm-based algorithms such as USPEX (Glass *et al.*, 2006 ▸; Oganov *et al.*, 2011 ▸; Lyakhov *et al.*, 2013 ▸), XtalOpt (Lonie & Zurek, 2011 ▸) and recent deep-learning-based models (Noh *et al.*, 2019 ▸; Ryan *et al.*, 2018 ▸). In practice, it is possible to generate random structures by forcibly combining different crystal structures *in silico*. The successful discovery of novel material structures under high pressure demonstrates the effectiveness of this approach when certain constraints can be set. However, it is not easy to rationally combine different crystal structures with different compositions and symmetry in a plausible manner. Therefore, oversight in the search for a small number of potential materials cannot be controlled. The combination of first-principles calculations and ML is required for creating effective methods for exploring materials.

One of the most common strategies for generating possible crystal structure candidates is to appropriately combine or apply the atomic substitution method to previously known structures. Beginning with a dataset of host crystal structures with known physical properties and predefined substitution operators, we can employ the atomic substitution method to create new hypothetical crystal structures with the same skeleton as that of the host crystal structure. Widely used substitution operators such as single-site, multisite or element substitution operators are selected depending on the host dataset and experts’ suggestions. These suggestions are typically based on domain knowledge about the physicochemical similarity between elements, atom–atom interactions, structural stability mechanisms and target physical property mechanisms. Consequently, the substitution method can work well with knowledge about material synthesis and lead directly to material synthesis ideas. Finally, an ‘understanding’ of the structure–stability relationship can be directly obtained from screening results, which can help in systematically correcting researchers’ suggestions.

### 1.1. Our contribution   

**Figure 1:**
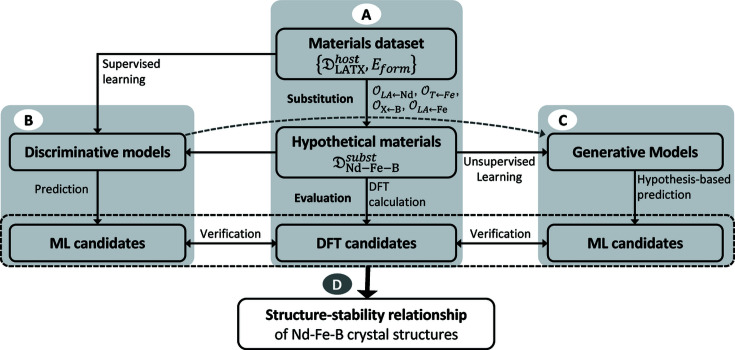
Workflow for extracting the structure–stability relationship of Nd–Fe–B crystal structures by integrating high-throughput first-principles calculations, supervised learning and unsupervised learning techniques.

In this study, we propose a protocol for exploring new crystal structures under a given combination of constituent elements and the use of data mining to elucidate the structure–stability relationship (Fig. 1 ▸). As a demonstration example, we search for the new crystal structures of Nd–Fe–B materials by applying the atomic substitution method to a dataset containing host crystal structures composed of lanthanides, transition metals and light elements. We apply high-throughput first-principles calculations (Fig. 1 ▸, block A) to estimate the formation energy. Based on this, we evaluate the phase stability (hereinafter referred to as stability) of all generated Nd–Fe–B crystal structures (Section 2.2). The new Nd–Fe–B structures discovered after the screening steps are presented in Section 2.3. Supervised models are trained to mimic first-principles calculations from the host and substitution crystal structures and their calculated formation energy. Based on results from supervised learning models, relevance analysis is performed to extract the hidden structural descriptors that determine the formation energy of the generated Nd–Fe–B crystal structures (Fig. 1 ▸, block B). Finally, we trained an unsupervised learning model (Fig. 1 ▸, block C) that uses the obtained relevant descriptors to appropriately group newly generated crystal structures. We compare the obtained group labels and potentially formable states of all crystal structures to determine the relationship between the structure and stability of the Nd–Fe–B crystal structures.

## 2. Screening for potential Nd–Fe–B crystal structures   

### 2.1. Creation of new crystal structure candidates   

In this study, we focus on crystalline magnetic materials comprising a lanthanide (*LA*), a transition metal (*T*) and a light element (*X*). We selected the *LA* atoms from Y, La, Ce, Pr, Nd, Pm, Sm, Eu, Gd, Tb, Dy, Ho, Er, Tm, Yb and Lu; *T* from Ti, V, Cr, Mn, Fe, Co, Ni, Cu, Zn, Y, Zr, Nb, Mo, Tc, Ru, Rh, Pd, Ag, Cd, Hf, Ta, W, Re, Os, Ir, Pt, Au and Hg; and *X* from H, B, C, N and O. We collected the details of 5967 well known crystal structures with formation energies from the Open Quantum Materials Database (OQMD) (Saal *et al.*, 2013 ▸) (version 1.1) to form the host material dataset, denoted 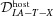. Each host crystal structure consists of one or two rare-earth metals, one or two transition metals and one light element. Additionally, from 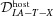 we selected a subset of all the crystal structures comprising Nd, Fe and B, denoted 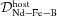.

We create new candidates for crystal structures consisting of Nd, Fe and B with the same skeleton as the host crystal structures in 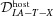 using a substitution method. For each host crystal structure, a substituted crystal structure is created by substituting all lanthanide sites with Nd, all transition metal sites with Fe and all light-element sites with B. The new structures are compared with each other and with the crystal structures in the 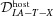 dataset to remove duplication. We follow the comparison procedure proposed by qmpy (the python application programming interface of OQMD) (Saal *et al.*, 2013*b* ▸). The structures of the materials are transformed into reduced primitive cells to compare the two lattices; all lattice parameters are compared. The internal coordinates of the structures are compared by examining all rotations allowed by each lattice and searching for rotations and translations to map the atoms of the same species into one another within a given level of tolerance. Here, any two structures in which the percentage deviation in lattice parameters and angles are smaller than 0.1 are considered to be identical. Furthermore, we apply our designed orbital field matrix (OFM) (Section 3.1) to eliminate duplication. Two structures are considered to be the same if the *L*_2_ norm of the difference in the OFM is less than 10^−3^. Note that two structures with the same shape but slightly different in size are considered to be identical. Finally, we obtain a dataset for the substituted crystal structures, denoted 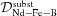, with 149 new non-optimized Nd–Fe–B crystal structures. These structures are then optimized using the first-principles calculations described in detail in Section 2.2.

### 2.2. Assessment of phase stability   

First-principles calculations based on DFT (Kohn & Sham, 1965 ▸; Hohenberg & Kohn, 1964 ▸) are one of the most effective calculation methods used in materials science. DFT calculations can accurately estimate the formation energy of materials, which is used to build phase diagrams for systems of interest. Hence, the phase stability of a material – in other words, the decomposition energy of a material (C—H distance) – is obtained via the convex hull analysis of phase diagrams and the decomposition of the material into other phases. We used the formation energy obtained from OQMD (Saal *et al.*, 2013*b* ▸; Kirklin *et al.*, 2015 ▸) of 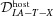 to build phase diagrams and calculate the C—H distance. The C—H distance of a material is defined as follows:
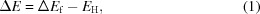
where Δ*E*_f_ is the formation energy and *E*_H_ is determined by projecting from the chemical composition position to an end point appearing on the convex hull facets. Details of the algorithm for finding these convex hull facets from hull points can be found in the work by Barber *et al.* (1996 ▸) and Saal *et al.* (2013 ▸). Hereafter, we consider the C—H distance Δ*E* as the degree of the phase stability of a material. A material that lies below or on the C—H surface, Δ*E* = 0, is a potentially formable material in nature, and a material associated with Δ*E* > 0 is unstable. A material associated with Δ*E* slightly above the C—H surface is considered to be in a metastable phase.

Metastable phases are synthesized in numerous cases, for which we consider a reasonable range for the C—H distance (Balachandran *et al.*, 2018 ▸). Referring to the prediction accuracy of formation energy [∼0.1 eV per atom by OQMD (Saal *et al.*, 2013 ▸)], we define all materials with Δ*E* ≤ 0.1 eV per atom as potentially formable structures and as unstable materials otherwise. Following this definition, 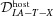 can be divided into subsets 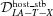 and 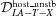 for potentially formable crystal structures and unstable crystal structures, respectively.


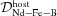
 includes 35 Nd–Fe–B crystal structures, which can be used as references to construct the Nd–Fe–B phase diagram. Seven materials were found for ternary materials, which were comprised of Nd, Fe and B. To verify the reliability of the dataset used to construct the phase diagram as well as the stability definition, we removed each ternary material and used the remaining materials in 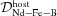 to estimate its corresponding convex hull distance. Under this test, among the seven ternary crystal structures, there are two formable ternary materials, NdFe_4_B_4_ and Nd_5_Fe_2_B_6_, which lie on the surface of the CH of the phase diagram with Δ*E* = 0.0. Additionally, one material, NdFe_12_B_6_, is potentially formable (metastable) with a stability of less than 0.1 eV per atom, as shown in Table VI of the supporting information. It should be noted that the important magnetic material, Nd_2_Fe_14_B, did not exist in the OQMD database at the time when we conducted this study. Based on the Nd–Fe–B phase diagram and the formation energy of −0.057 eV per atom calculated using DFT, the corresponding Δ*E* ^DFT^ is 1.4 × 10^−4^ eV per atom. This result implies that Nd_2_Fe_14_B is in the stable phase. To conclude, we confirm that the experimentally synthesized structures all satisfy the stability definition given in equation (1) in this section.

We followed the computational settings of OQMD (Saal *et al.*, 2013*b* ▸; Kirklin *et al.*, 2015 ▸) for estimating the formation energy of the newly created Nd–Fe–B crystal structures in 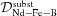. The calculations were performed using the *Vienna Ab initio Simulation Package* (VASP) (Kresse & Hafner, 1993 ▸, 1994 ▸; Kresse & Furthmüller, 1996*a* ▸,*b* ▸) by utilizing projector-augmented wave method potentials (PAW) (Blöchl, 1994 ▸; Kresse & Joubert, 1999 ▸) and the Perdew–Burke–Ernzerhof (PBE) (Perdew *et al.*, 1996 ▸) exchange-correlation functional.

We employed DFT + U for Fe, and all calculations were spin-polarized with ferromagnetic alignment of the spins and with initial magnetic moments of 5, 0 and 0 μ_B_ for Fe, Nd and B, respectively. For each newly created structure, we performed coarse optimization, fine optimization and a single-point calculation, following the ‘coarse relax’, ‘fine relax’ and ‘standard’ procedures of the OQMD. The *k*-grid for these calculation series is selected by the *k*-points per reciprocal atom (KPRA): 4000, 6000 and 8000 for ‘coarse relax’, ‘fine relax’ and ‘standard’, respectively. We used a cutoff energy of 520 eV for all calculations. The total energies of the standard calculations are used for the formation energy calculations, 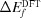. The C—H distance of a newly created structure can be estimated from 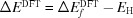.

**Figure 2:**
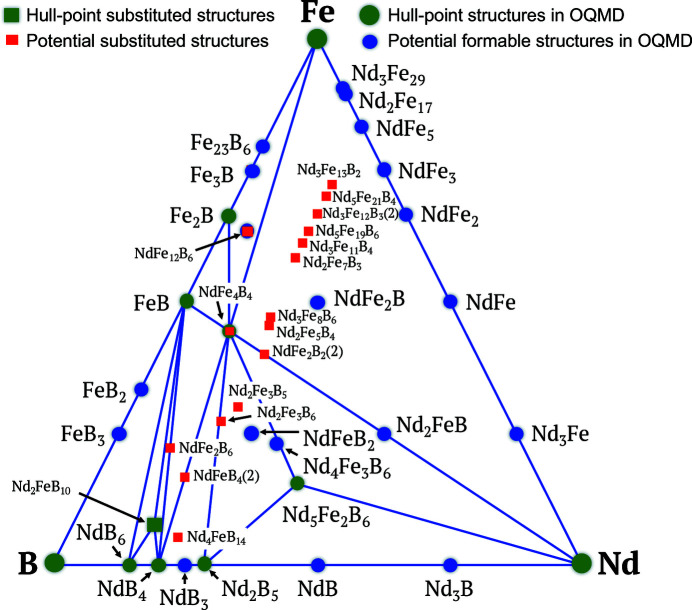
Phase diagram of Nd–Fe–B including materials obtained from OQMD (blue circles) and 20 new substituted structures confirming it is potentially formable (red squares). Hull points are denoted in green. The total number of disparate structures with the same chemical composition is shown in parentheses.

After calculating the formation energy, we found 20 new Nd–Fe–B crystal structures that are not in 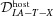, in which the C—H distance of the corresponding optimized structure is less than 0.1 eV. These structures originate from different host structures with different skeletons. Note that we found one structure, Nd_2_FeB_10_, with a stability of less than −0.01 eV per atom. Thus, this structure is also used as a reference to construct the Nd–Fe–B phase diagram. Among the 20 new Nd–Fe–B structures, there are three pairs of indistinguishable structures sharing the same chemical compositions (NdFe_2_B_2_, NdFeB_4_ and NdFe_4_B). Details about these structures are given in Table 1 ▸. The phase diagram of the Nd–Fe–B materials, including the 20 new substituted structures, is shown in Fig. 2 ▸.

We also calculated the magnetization of these materials. We used open-core approximation to treat the 4*f* electrons of Nd. The contribution of 4*f* electrons to the magnetization is 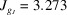. The magnetization is normalized to the volume of a unit cell:
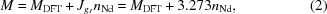
where *M*_DFT_ is the magnetization given by DFT and *n*_Nd_ is the number of Nd atoms in the unit cell. All calculation results are summarized in Table 1 ▸.

### 2.3. Newly discovered Nd–Fe–B crystal structures   

**Figure 3:**
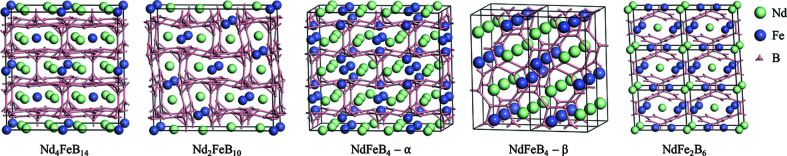
Representative Nd–Fe–B structures discovered by applying the elemental substitution method to the lanthanide, transition metal and rare-earth material dataset. Left to right: Nd_4_FeB_14_, Nd_2_FeB_10_, NdFeB_4_-α, NdFeB_4_-β and NdFe_2_B_6_. All 20 structures discovered are shown in the supporting information.

Fig. 3 ▸ shows five specific crystal structures of the predicted formable crystal structures. A common characteristic of these crystal structures is that boron atoms form a network structure and Nd and Fe atoms are surrounded by the cages formed by the boron atom network. In the Nd_4_FeB_14_ crystal structure, these boron cages are arranged in parallel and Fe atoms are sandwiched between two halves of the boron atom octahedron. In the crystal structure of Nd_2_FeB_10_, which is confirmed by DFT calculations and selected as the hull point in the phase diagram, Nd and Fe atoms are trapped in the boron atom cages; however, these cages are arranged in herringbone patterns. Interestingly, two stable crystal structures of NdFeB_4_ were found as the proportion of Fe increased. One NdFeB_4_-α structure was obtained by the elemental substitution of the original CeNiB_4_ crystal structure [id: 2023354 (Akselrud *et al.*, 1984 ▸)]. This crystal structure is similar to the Nd_4_FeB_14_ crystal structure, with cages formed by boron networks that trap Nd and Fe atoms and are arranged in parallel. In contrast, in the other predicted crystal structure for NdFeB_4_ {NdFeB_4_-β structure obtained by the elemental substitution of the CeCrB_4_ [id: 2023373 (Kuzma *et al.*, 1973 ▸)] crystal structure}, the boron atoms form a planar network structure comprised of heptagon–pentagon ring pairs. Another form of boron cage is found in the NdFe_2_B_6_ crystal structure. All potentially formable crystal structures are shown in detail in the supporting information.

## 3. Mining structure–stability relationship of Nd–Fe–B crystal structures   

### 3.1. Materials representation   

We must convert the information regarding the materials into descriptor vectors. We employ the OFM (Lam Pham *et al.*, 2017 ▸; Pham *et al.*, 2018 ▸) descriptor with a minor modification. The OFM descriptors are constructed using the weighted product of the one-hot vector representations, 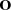, of atoms. Each vector 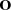 is filled with zeros, except those representing the electronic configuration of the valence electrons of the corresponding atom. The OFM of a local structure, named θ, is defined as follows:
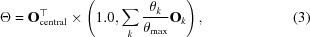
where θ_*k*_ is the solid angle determined by the face of the Voronoi polyhedra between the central atom and the index *k* neighboring atom, and θ_max_ is the maximum solid angle between the central atom and neighboring atoms. By removing the distance dependence in the original OFM formulation (Lam Pham *et al.*, 2017 ▸; Pham *et al.*, 2018 ▸), we focus exclusively on the coordination of valence orbitals and the shape of the crystal structures. The mean over the local structure descriptors is used as the descriptor of the entire structure:
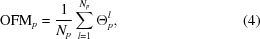
where *p* is the structure index, and *l* and *N*_*p*_ are the local structure indices and the number of atoms in the unit cell of the structure *p*, respectively.

Note that owing to the designed cross product between the atomic representation vectors of each atom, each element in the matrix represents the average number of atomic coordinates for a certain type of atom. For example, an element of a descriptor obtained by considering the product of a *d* ^6^ element of the center atom representation and an *f* ^4^ element of the environment atom representation, denoted (*d* ^6^, *f* ^4^), shows an average coordination number of *f* ^4^ (Nd) sites surrounding all *d* ^6^ (Fe) sites. As the term *s* ^2^ appears at all descriptors for Fe, Nd and B sites, the element (*s* ^2^, *s* ^2^) represents the average coordination number of a given structure. All of these OFM elements provide a foundation for the intuitively interpretable investigation of the structure–stability relationship.

### 3.2. Mining of formation energy data of *LA*–*T*–*X* crystal structures with a supervised learning method   

We trained the ML models that can predict the formation energy of the crystal structures, Δ*E*_f_, from 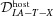, which is represented using the OFM descriptor and the corresponding known formation energy data. We applied kernel ridge regression (KRR) (Murphy, 2012 ▸), which is demonstrated to be useful for predicting material properties. In the KRR algorithm, the target variable, *y* = Δ*E*_f_, is represented by a weighted kernel function as follows:
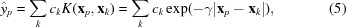
where 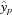 is the predicted formation energy of crystal structure *p*; **x**_*p*_ and **x** *_k_* are the representation vectors of crystal structures *p* and *k* based on the OFM descriptor, respectively; *k* runs over all crystal structures in the training set; 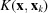 is the Laplacian kernel function. The *c_k_* coefficients are estimated by minimizing the total square error regularized by the *L*_2_ norm as follows: 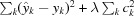, where *y_k_* and 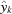 are the observed and predicted target values of the structure *k*, respectively. We perform a ten-times tenfold cross-validation process to determine parameters λ and γ in the KRR models. These parameters are selected by minimizing the mean absolute error (MAE) of the validation set.

**Figure 4:**
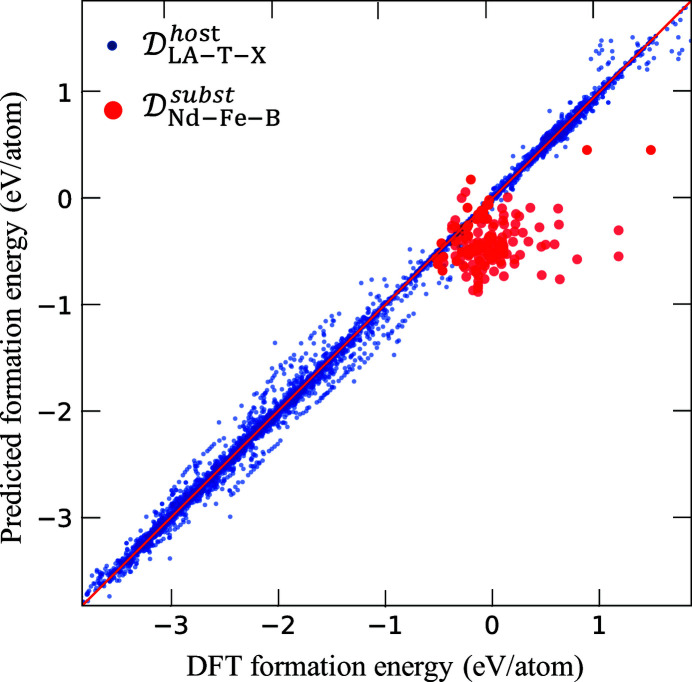
Comparison of formation energies calculated using DFT and those predicted through ML using the KRR model with the OFM descriptor. The blue and red solid circles represent the cross-validated results for 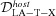 and the prediction results for 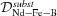, respectively.

Fig. 4 ▸ shows the ten-times tenfold cross-validated comparison of the formation energies calculated using DFT and those predicted by the KRR model for the crystal structures in 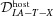 (blue circles). Fig. 4 ▸ also shows a comparison of the formation energies calculated using DFT and those predicted using the KRR model (trained using all crystal structures in 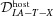) for the crystal structures in 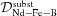 (red circles). In the cross-validated comparison of materials in 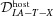, the formation energies predicted via KRR show good agreement with those calculated using DFT, with an *R* ^2^ (Kvålseth, 1985 ▸) value of 0.990 (1), see Table 2 ▸.

It should be noted that this predictive model is learned from the data (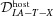) containing only the optimized crystal structures. Thus, when applied to a newly generated non-optimized crystal structure (in 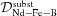), it is clear that the possibility of correctly predicting the formation energy is low. The MAE of the KRR-predicted formation energy of the crystal structures in 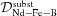 after structure optimization is approximately 0.3 (eV per atom), which is three times larger than the cross-validated MAE result. The results of applying the KRR prediction model to estimate the stability of these hypothetical materials are shown in detail in Section 3.5.

### 3.3. Descriptor-relevance analysis   

Furthermore, we focus on 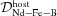 and evaluate the relevance (Nguyen *et al.*, 2019 ▸; Yu & Liu, 2004 ▸; Visalakshi & Radha, 2014 ▸) of each element in the OFM descriptor with respect to the formation energy of the crystal structure. We utilize the change in prediction accuracy when removing or adding a descriptor [from the full set of descriptors (Nguyen *et al.*, 2018 ▸) in the OFM] to search for the descriptors that are strongly relevant (Nguyen *et al.*, 2019 ▸; Dam *et al.*, 2018 ▸) to the formation energy (*i.e.* C—H distance and phase stability) of the Nd–Fe–B crystal structures.

In detail, for a given set *S* of descriptors, we define the prediction capacity *PC*(*S*) of *S* by the maximum prediction accuracy that the KRR model can achieve using the variables in a subset *s* of *S* as follows:
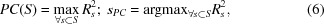
where 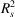 is the value of the coefficient of determination *R* ^2^ (Kvålseth, 1985 ▸) achieved by the KRR using a set *s* as the independent variables. *s_PA_* is the subset of *S* that yields the prediction model having the maximum prediction accuracy.

**Figure 5:**
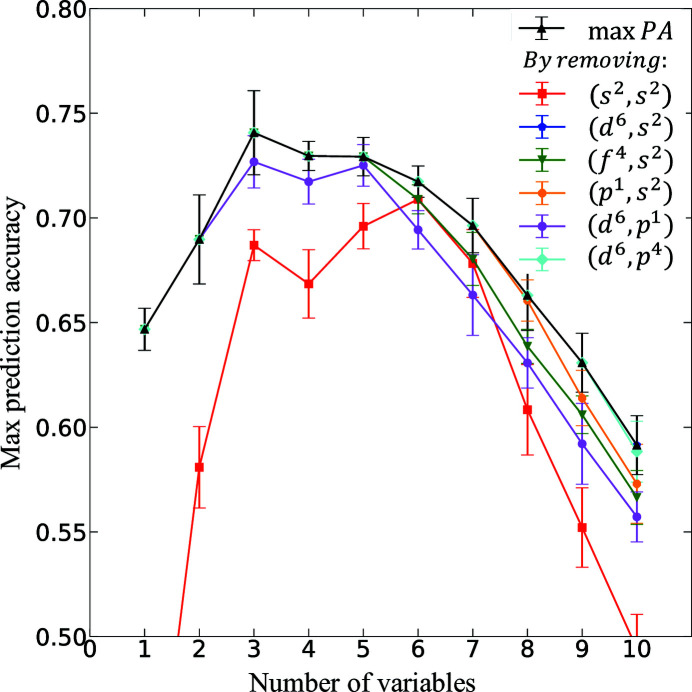
Results of the relevance analysis performed for predicting *E*_f_ of all Nd–Fe–B materials present in 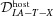. By removing the descriptor (*s* ^2^, *s* ^2^), the maximum prediction capacity (red line) is significantly reduced compared with the maximum prediction capacity line (max. *PC*) of all descriptor sets (black line).

**Figure 6:**
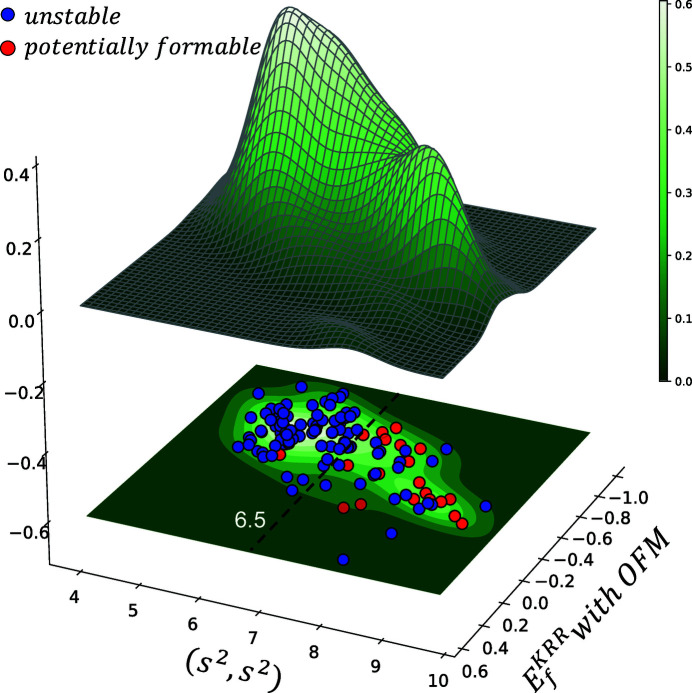
Distribution of substituted materials in space with the *x* axis showing the KRR-predicted formation energy, 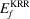, with non-optimized structures and the *y* axis showing the extracted strongly relevant descriptor (*s* ^2^, *s* ^2^). The black dotted line shows the limitation of (*s* ^2^, *s* ^2^), which maximizes the separation between two mixture distributions.

Let *S_i_* denote a set of descriptors after removing a descriptor *x_i_* from the full descriptor set *S*; *S_i_* = S − {*x_i_*}. A descriptor is strongly relevant if and only if
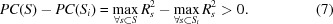
Fig. 5 ▸ summarizes the results obtained from the descriptor-relevance analysis. The black-triangled curve shows the dependence of the maximum prediction capacity (max. *PC*, in *R* ^2^ score) on the number of variables/OFM descriptors used in regression models. Other curves show the dependence of the maximum prediction capacity on the number of OFM descriptors used in regression models when a specific OFM is removed from the whole set of OFM descriptors. For example, the orange-dotted curve illustrates the max. *PC* of the OFM descriptor set without the appearance of the (*p* ^1^, *s* ^2^) descriptor. It is evident that the descriptor (*s* ^2^, *s* ^2^) (red-squared curve) is highly relevant to the prediction of the formation energy of the crystal structures in 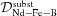. For further investigation, we project all substituted crystal structures in 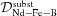 into the space of the KRR-predicted formation energy, 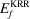 and (*s* ^2^, *s* ^2^), as shown in Fig. 6 ▸. One can easily deduce that the distribution of 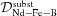 is a mixture of two distribution components. The larger distribution component is located in the region (*s* ^2^, *s* ^2^) < 6.5, whereas the other is located in the region (*s* ^2^, *s* ^2^) ≥ 6.5. We infer the existence of two distinct groups of substituted crystal structures. The first group contains structures with average atomic coordination numbers lower than 6.5, and the second group contains structures with average atomic coordination numbers higher than 6.5. Furthermore, most newly discovered potentially formable crystal structures belong to the second group.

### 3.4. Mining of substituted Nd–Fe–B crystal structure data with an unsupervised learning method   

In this section, we demonstrate the use of the proposed generative model, which applies the relevance analysis results and unsupervised learning, in contrast to the conventional supervised learning approach. As a result, this model performs detailed investigations at particular sites whose coordination numbers are highly correlated to the structure–stability relationship.

The underlying hypothesis of this approach is that there are various correlation patterns between crystal structure properties and their formation energies. Naturally, most of these patterns are for unstable crystal structures and only a few of these pertain to potentially formable crystal structures. These patterns might not be exposed directly through the feature-relevance analysis method due to the multivariate correlation between the target and description variables. The strong relevant descriptor (*s* ^2^, *s* ^2^) can appear as an extracted pattern to indicate the correlation between the structure–stability relationship. As the term *s* ^2^ appears at all descriptors for Fe, Nd and B sites, (*s* ^2^, *s* ^2^) indicates only the average atomic coordination numbers, which do not precisely represent the coordination number of any particular site. On the contrary, other OFM descriptors are designed to explicitly represent the coordination number of all pairwise elements. As the two terms *d* ^6^ and *f* ^4^ appear at only descriptors for Fe or Nd, respectively, in order to investigate the average coordination number of the Fe, Nd and B sites, in addition to (*s* ^2^, *s* ^2^), we focus on the values of the descriptors (*d* ^6^, *s* ^2^) and (*f* ^4^, *s* ^2^). These descriptors represent the average atomic coordination numbers of Fe sites and Nd sites. Furthermore, we also focus on the values of the OFM descriptors (*d* ^6^, *d* ^6^), (*d* ^6^, *f* ^4^), (*f* ^4^, *d* ^6^) and (*f* ^4^, *f* ^4^). These descriptors represent the average number of Fe sites surrounding the Fe sites, Nd sites surrounding the Fe sites, Fe sites surrounding the Nd sites and Nd sites surrounding the Nd sites. These OFM descriptors are useful in discussing not only the structure–stability relationship but also the strength of magnetic-exchange couplings between the 3*d* orbitals of Fe and the 4*f* orbitals of Nd.

**Figure 7:**
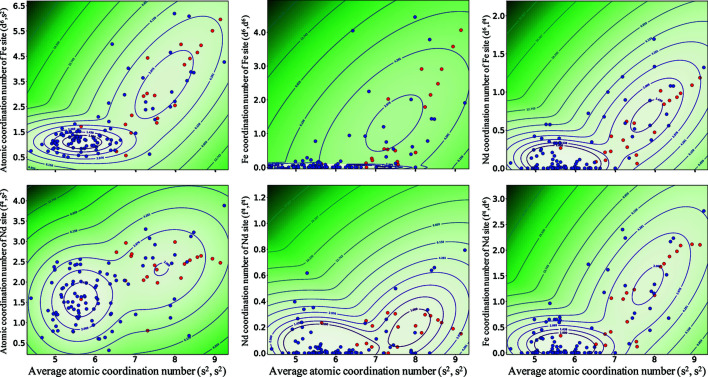
Density distribution of the newly generated Nd–Fe–B crystal structures in two-dimensional space obtained using selected OFM descriptors. The blue and red solid circles represent the unstable and potentially formable crystal structures verified by DFT calculations, respectively. Contour lines show the isodense surface of the distribution.

Fig. 7 ▸ shows the density distribution of the newly created crystal structures, 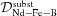, in two-dimensional space using the selected descriptors. For all pairs of descriptors, the density distribution is similar to the distribution of (*s* ^2^, *s* ^2^) and 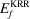 shown in Fig. 6 ▸ with two clear peaks, one large and one small, with slight overlap. This result again confirms that (*s* ^2^, *s* ^2^) is highly relevant for expressing the nature of the distribution of the newly created crystal structures. In addition, (*d* ^6^, *s* ^2^) and (*d* ^6^, *d* ^6^) are important for identifying the characteristics of the distribution. It should be noted that these features could not be exposed using feature-relevance analysis since the prediction model can utilize the information from other highly correlated features instead, *e.g.* (*s* ^2^, *s* ^2^). In contrast, the average coordination number of the Nd sites (*f* ^4^, *s* ^2^) and the average coordination number of the Nd sites around the Nd sites (*f* ^4^, *f* ^4^) have a weak relationship with the characteristics related to the distribution of these crystal structures. These results indicate that the average coordination number of the Fe sites (*d* ^6^, *s* ^2^) and the average coordination number of the Fe sites around the Fe sites (*d* ^6^, *d* ^6^) are extremely important for characterizing the newly created Nd–Fe–B crystal structures.

We employed a GMM (Murphy, 2012 ▸) for learning the patterns of crystal structures by clustering 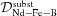 into groups. The GMM model is based on the assumptions that the data consist of different groups and the data in each group follow their own Gaussian distribution. In other words, in the GMM, the distribution of data are fitted to a combination of a certain number, *M*, of Gaussian functions  (Murphy, 2012 ▸) where *M* represents the number of data groups. The probability distribution of a crystal structure with index *p*, represented using selected descriptors, **x**_*p*_ and *f*(**x**_*p*_), can be approximated as follows:
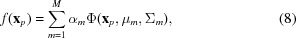
where
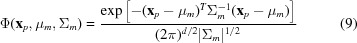
is a multivariate Gaussian distribution with mean μ_*m*_ and covariance matrix Σ_*m*_ and *d* is the dimension of the representation vector **x**_*p*_. The α_m_ coefficients are the weights that satisfy the following constraint:
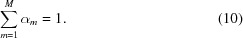
The probability that **x**_*p*_ belongs to group *m* can be represented as follows:
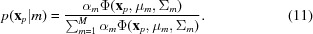
The model parameters α_*m*_, μ_*m*_ and Σ_*m*_ are determined using an expectation-maximization algorithm (Pedregosa *et al.*, 2011 ▸). The number of data groups, *M*, is fixed at two in this study. It is interesting to note that the GMM provides a ‘probabilistic image’ of the pattern of crystal structures, wherein it provides the probability of a crystal structure remaining in a group instead of assigning the crystal structures to a specific group. The sum of the probabilities of crystal structures remaining in either of the groups is one. Therefore, the GMM is expected to discover distinctive patterns of crystal structures from the data and calculate the probability that a crystal structure belongs to a group.

We can label the newly generated crystal structures by fitting the data 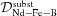 to the GMM with two Gaussian distributions and calculating the probabilities of the crystal structures belonging to each group. Given that it is not easy to find a new potential formable crystal structure, we suppose that most newly generated structures are unstable and only a few are potentially formable. Therefore, we infer that the large Gauss component corresponds to the distribution of unstable crystal structures and the small Gauss component corresponds to the distribution of potential formable crystal structures. This hypothesis can be verified through comparison with the results of the DFT calculations, and it can be seen that most of the potential formable crystal structures confirmed by DFT calculation actually belong to the small Gauss component. This implies that the phase stabilities of the Nd–Fe–B crystals are not significantly related to the coordination number of the Nd sites but are largely determined by the coordination number of the Fe sites, suggesting that, if the Nd sites can be replaced in part by Fe, the crystal structure characteristics of Nd–Fe–B which are directly related to its phase stability can be controlled. Further application of this discovery in the design of Nd–Fe–B crystal materials is promising.

### 3.5. Learning prediction models for the phase stability of crystal structures   

A large number of ML applications reported to date (in materials science research) state the effectiveness and applicability of ML methods using statistical tests (such as cross validation). However, statistical tests are methods for assessing the risk in predicting the physical properties of the most optimized-structure materials, and are not appropriate for predicting and discovering novel materials. Therefore, in this study, to verify whether ML techniques are effective in searching for new potentially formable Nd–Fe–B crystal structures, we trained three supervised ML models from 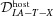 and one unsupervised model from 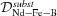. In addition, we tested whether the models can predict the stability of the newly created crystal structures in 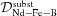. The three supervised ML models are trained by considering 5967 materials in 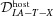 with the OFM descriptor and the stability target values described in Sections 3.1 and 2.2. Then, all models are applied to predict the 149 newly hypothetical structures in 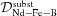 while considering the stability calculated by the DFT as references in prediction accuracy evaluation.

In the first model (KRR model), the C—H distance is calculated using the formation energy predicted by the KRR model described in Section 3.2. Then, we applied a threshold of 0.1 eV per atom to the obtained C—H distance to determine whether the crystal structure is potentially formable. It is worth noting again that the bottleneck of this method is that the formation energy prediction model is learned from data containing only the optimal crystal structures. Therefore, the formation energy is not predicted correctly when the method is applied to a newly created non-optimal crystal structure.

The second model is a logistic regression model (LG model). From the two subsets of 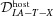, including the potentially formable (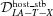) and unstable (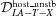) crystal structures, we modelled the probability of observing potentially formable (*y* = 1) and unstable (*y* = 0) class labels directly using classification models. We hypothesized that the probability of observing potentially formable materials, 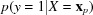, follows:
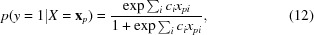
where **x**_*p*_ is the description vector of structure *p* (obtained by flattening the OFM), *i* is the index of vector elements in **x**_*p*_ and *c_i_* is the coefficient of the corresponding element *x*_π_. In our experiments, all *c_i_* coefficients are obtained via maximum *a posteriori* estimation using *L*_1_ as the regularization term (Ng, 2004 ▸; Lee *et al.*, 2006 ▸). The third model is the decision tree model (DT model) (Murphy, 2012 ▸), which uses information gain (Breiman *et al.*, 1984 ▸; Hastie *et al.*, 2009 ▸) as the criterion to measure the quality of tree-splitting.

The unsupervised model is based on the observations of the mixture distribution of the newly created crystal structures, 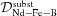. We build the fourth model (GMM) by assuming that the major and minor Gauss components obtained correspond to the ‘unstable’ and ‘potentially formable’ class labels of the crystal structures, respectively.

The evaluation results of the four models are summarized in Table 3 ▸. We use three evaluation scores: *Precision*, *Recall* and *f*_1_. The *Precision* score (also referred to as positive predictive value) with respect to the unstable structure class is the fraction of the unstable crystal structures predicted correctly among the number of crystal structures predicted to be unstable (Perry *et al.*, 1955 ▸). The *Recall* score (also known as sensitivity) with respect to the unstable structure class is the fraction of the unstable crystal structures predicted correctly among all crystal structures that are actually unstable (Perry *et al.*, 1955 ▸). The *Precision* and *Recall* scores are combined in the *f*_1_ score (or *f*-measure) to provide a single measurement (Derczynski, 2016 ▸). To compare the classification ability of ML models, we summarize the evaluation scores of all classes (*i.e.* ‘unstable’ and ‘potentially formable’) by utilizing a macro averaging method (Su *et al.*, 2015 ▸) which is implemented in *sklearn.metrics.average_precision_score* (version 0.21.3; Pedregosa *et al.*, 2011 ▸).

The KRR model shows the lowest values of all evaluation scores among the three supervised learning models where *Precision*, *Recall* and *f*_1_ are 0.533, 0.534 and 0.376, respectively. In contrast, the DT model provides the most accurate prediction. This model accurately predicts the potentially ‘formable unstable’ label of all substituted Nd–Fe–B crystal structures with 0.704 macro *Precision* score and obtains macro *Recall* and *f*_1_ scores of 0.676 and 0.687, respectively. The LG model shows the highest macro *Recall* score, 0.687, compared with the other two supervised learning models.

The final but most surprising result is that the unsupervised GMM is superior to the other three supervised learning models in all three evaluation scores. The average *Precision* and *Recall* scores of the GMM are 0.729 and 0.821, respectively, which are significantly higher than those of the three supervised learning models. This result shows that the integration of descriptor-relevance analysis and unsupervised learning with the GMM is superior to conventional ML models, such as KRR, LG and DT, for obtaining information about the phase stability of substituted Nd–Fe–B crystal structures. We also investigated the usefulness of ensembling models. As the prediction problem under consideration is a binary classification, we implement two well known operators, ‘AND’ and ‘OR,’ for combining classification results. The details of the results are shown in Tables 4 ▸ and 5 ▸. These results again suggest that the structure–stability relationship obtained using data mining is highly promising for the design of Nd–Fe–B materials.

## 4. Conclusions   

We focus on discovering new Nd–Fe–B materials using the elemental substitution method with *LA*–*T*–*X* compounds, with a lanthanide, transition metal and light element (*X* = B, C, N, O) as host materials. For each host crystal structure, a substituted crystal structure is created by substituting all lanthanide sites with Nd, all transition metal sites with Fe and all light-element sites with B. High-throughput first-principles calculations are applied to evaluate the phase stability of the newly created crystal structures, and twenty of them are found to be potentially formable. We implemented an approach by incorporating supervised and unsupervised learning techniques to estimate the stability and analyze the relationship between the structure and stability of the newly created Nd–Fe–B crystal structures. Three supervised learning models (KRR, LG and DT) learned from *LA*–*T*–*X* host crystal structures achieved the maximum accuracy and *Recall* scores of 70.4 and 68.7%, respectively, in predicting the stability state of new substituted Nd–Fe–B crystals. The proposed unsupervised learning model resulting from the integration of descriptor-relevance analysis and the GMM provides accuracy and *Recall* scores of 72.9 and 82.1%, respectively, which are significantly better than those of the supervised models. Moreover, the unsupervised learning model can capture and interpret the structure–stability relationship of the Nd–Fe–B crystal structures. The average atomic coordination number and the coordination number of the Fe sites are quantitatively shown to be the most important factors in determining the phase stability of the new substituted Nd–Fe–B crystal structures.

## Supplementary Material

Supporting tables. DOI: 10.1107/S2052252520010088/yu5018sup1.pdf

## Figures and Tables

**Table 1:** Properties of new Nd–Fe–B materials: formation energy by DFT 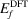 (eV per atom), stability by DFT 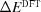, magnetization *M* (μ_B_ per formula unit and μ_B_ Å^−3^ in parentheses) and mean displacement Δ*r*, estimated by hypothesized structures and final-optimized structures

Formula	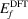 (eV per atom)	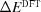 (eV per atom)	*M* [μ_B_ (μ_B_ Å^−3^)]	Δ*r* (Å)	Host materials	OQMD id of host materials
Nd_2_FeB_10_	−0.522	−0.011	13.11 (0.050)	0.038	Ce_2_NiB_10_	2025052 (Jeitschko *et al.*, 2000 ▸)
NdFe_2_B_6_	−0.473	0.008	3.30 (0.040)	0.150	CeCr_2_B_6_	94775 (Kuzma & Svarichevskaya, 1972 ▸)
Nd_4_FeB_14_	−0.506	0.030	26.30 (0.063)	0.069	Ho_4_NiB_14_	2107958 (Geupel *et al.*, 2001 ▸)
NdFe_2_B_2_-α	−0.343	0.046	4.41 (0.067)	0.085	DyCo_2_B_2_	1852452 (Niihara *et al.*, 1987 ▸)
NdFeB_4_-α	−0.462	0.052	17.42 (0.073)	0.041	CeNiB_4_	2023354 (Akselrud *et al.*, 1984 ▸)
NdFeB_4_-β	−0.455	0.060	18.73 (0.072)	0.050	CeCrB_4_	2023373 (Kuzma *et al.*, 1973 ▸)
Nd_2_Fe_3_B_5_	−0.374	0.066	6.85 (0.055)	0.143	Eu_2_Os_3_B_5_	180411 (Schweitzer & Jung, 1986 ▸)
Nd_2_Fe_5_B_4_	−0.284	0.069	10.31 (0.077)	0.206	Eu_2_Rh_5_B_4_	183842 (Jung, 1990 ▸)
NdFe_4_B-α	−0.092	0.070	21.64 (0.134)	1.769	CeCo_4_B	185365 (Kuzma & Bilonizhko, 1973*a* ▸)
NdFe_12_B_6_	−0.231	0.072	45.56 (0.117)	1.012	CeNi_12_B_6_	2077072 (Akselrud *et al.*, 1985 ▸)
Nd_5_Fe_21_B_4_	−0.052	0.077	57.73 (0.140)	2.342	Nd_5_Co_21_B_4_	126928 (Liang *et al.*, 2001 ▸)
Nd_5_Fe_19_B_6_	−0.115	0.080	50.02 (0.128)	1.820	Nd_5_Co_19_B_6_	125302 (Liang *et al.*, 2001 ▸)
NdFe_4_B-β	−0.081	0.081	65.19 (0.135)	0.241	NdNi_4_B	2069928 (Salamakha *et al.*, 2003 ▸)
Nd_3_Fe_13_B_2_	−0.027	0.081	36.12 (0.144)	2.961	Ce_3_Ni_13_B_2_	1778822 (Kuzma, 1981 ▸
Nd_3_Fe_11_B_4_	−0.131	0.085	28.22 (0.122)	0.150	Ce_3_Co_11_B_4_	1852403 (Kuzma & Bilonizhko, 1973*b* ▸)
Nd_2_Fe_3_B_6_	−0.375	0.088	16.02 (0.066)	0.132	Ce_2_Re_3_B_6_	1966804 (Kuzma *et al.*, 1989 ▸)
NdFe_4_B_4_	−0.342	0.090	17.30 (0.048)	0.140	CeRu_4_B_4_	2074891 (Poettgen *et al.*, 2010 ▸)
NdFe_2_B_2_-β	−0.297	0.092	7.25 (0.057)	0.142	CeIr_2_B_2_	180315 (Jung, 1991 ▸)
Nd_3_Fe_8_B_6_	−0.249	0.094	16.06 (0.079)	0.543	Eu_3_Rh_8_B_6_	1771853 (Jung, 1990 ▸)
Nd_2_Fe_7_B_3_	−0.147	0.096	35.04 (0.116)	0.209	Ce_2_Co_7_B_3_	2016489 (Kuzma & Bilonizhko, 1974 ▸)

**Table 2:** Ten-times tenfold cross-validation results provided by the KRR model in predicting formation energy

Model	*R* ^2^	MAE (eV per atom)	RMSE (eV per atom)
Kernel ridge	0.990 (1)	0.094 (2)	0.137 (1)

**Table 3:** Evaluation results of KRR, LG and DT models, and unsupervised GMM in estimating the stability of materials in 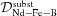

Model	*Precision*	*Recall*	*f*_1_
KRR model	0.533	0.534	0.376
LG model	0.629	0.687	0.599
DT model	0.704	0.676	0.687
GMM	0.729	0.821	0.735

**Table 4:** Classification results in predicting the ‘potentially formable’ class label of substituted materials with KRR, LG and DT models, GMM, and ensemble models The AND and OR operators in these ensemble models are denoted by & and |, respectively.

	KRR	LG	DT	GMM	KRR | GMM	LG | GMM	DT | GMM	KRR & GMM	LG & GMM	DT & GMM
*Precision*	0.24	0.35	0.56	0.49	0.24	0.36	0.48	**0.58**	0.53	**0.58**
*Recall*	0.82	0.79	0.45	0.91	0.97	**1.0**	0.91	0.76	0.7	0.45
*f*_1_	0.37	0.49	0.5	0.64	0.39	0.53	0.63	**0.66**	0.61	0.51

**Table 5:** Classification results in predicting the ‘unstable’ class label of substituted materials with KRR, LG and DT models, GMM, and ensemble models The AND and OR operators in these ensemble models are denoted by & and |, respectively.

	KRR	LG	DT	GMM	KRR | GMM	LG | GMM	DT | GMM	KRR & GMM	LG & GMM	DT & GMM
*Precision*	0.83	0.91	0.85	0.97	0.94	**1.0**	0.97	0.92	0.91	0.85
*Recall*	0.25	0.59	0.90	0.73	0.14	0.49	0.72	0.84	0.83	**0.91**
*f*_1_	0.38	0.71	0.87	0.83	0.24	0.66	0.83	**0.88**	0.86	**0.88**
