# Supplementary material for: Explainable machine learning for materials discovery: predicting the potentially formable Nd–Fe–B crystal structures and extracting the structure–stability relationship
Source: IUCrJ. 2020 Sep 23;7(Pt 6):1036–47. doi: 10.1107/S2052252520010088 (PMC7642775; doi:10.1107/S2052252520010088)
Supplement: Supplementary file 1 [file m-07-01036-sup1.pdf]

IUCrJ

Volume 7 (2020)

Supporting information for article:

Explainable machine learning for materials discovery: predicting the potentially formable Nd–Fe–B crystal structures and extracting the structure–stability relationship

Tien-Lam Pham, Duong-Nguyen Nguyen, Minh-Quyet Ha, Hiori Kino, Takashi Miyake and Hieu-Chi Dam

SUPPLEMENTAL MATERIALS

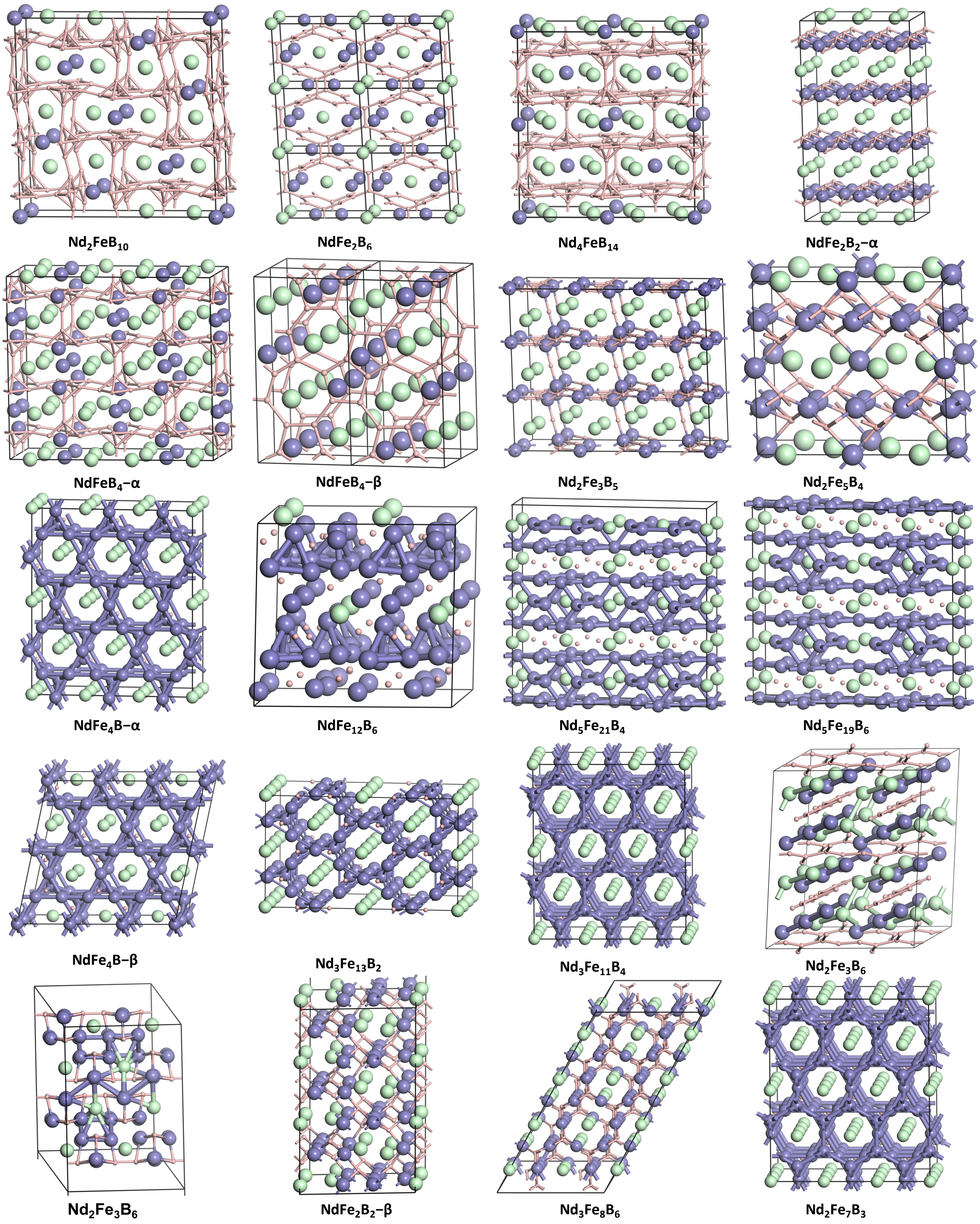

FIG. 8. Twenty potentially formable Nd-Fe-B structures extracted by applying elemental substitution method to lanthanide–transition metal–light element materials.

TABLE I. Properties of new Nd-Fe-B materials: formation energy by DFT E_f^{DFT} (eV/atom), stability by DFT ΔE^{DFT}, magnetization M (μ_B per formula unit and μ_B per Å^3 in parentheses), and mean displacement Δr, estimated by hypothesized structures and final-optimized structures.

Formula	E_f^{DFT} (eV/atom)	ΔE^{DFT} (eV/atom)	M (μ_B (μ_B/Å^3))	Δr (Å)	Host materials	OQMD id of host materials
Nd_2FeB_{10}	-0.522	-0.011	13.11 (0.050)	0.038	Ce_2NiB_{10}	2025052 [35]
$NdFe_2B_6$	-0.473	0.008	3.30 (0.040)	0.150	$CeCr_2B_6$	94775 [36]
Nd_4FeB_{14}	-0.506	0.030	26.30 (0.063)	0.069	Ho_4NiB_{14}	2107958 [37]
$NdFe_2B_2-\alpha$	-0.343	0.046	4.41 (0.067)	0.085	$DyCo_2B_2$	1852452 [38]
$NdFeB_4-\alpha$	-0.462	0.052	17.42 (0.073)	0.041	$CeNiB_4$	2023354 [39]
$NdFeB_4-\beta$	-0.455	0.060	18.73 (0.072)	0.050	$CeCrB_4$	2023373 [40]
$Nd_2Fe_3B_5$	-0.374	0.066	6.85 (0.055)	0.143	$Eu_2Os_3B_5$	180411 [41]
$Nd_2Fe_5B_4$	-0.284	0.069	10.31 (0.077)	0.206	$Eu_2Rh_5B_4$	183842 [42]
$NdFe_4B-\alpha$	-0.092	0.070	21.64 (0.134)	1.769	$CeCo_4B$	185365 [43]
$NdFe_{12}B_6$	-0.231	0.072	45.56 (0.117)	1.012	$CeNi_{12}B_6$	2077072 [44]
$Nd_5Fe_{21}B_4$	-0.052	0.077	57.73 (0.140)	2.342	$Nd_5Co_{21}B_4$	126928 [45]
$Nd_5Fe_{19}B_6$	-0.115	0.080	50.02 (0.128)	1.820	$Nd_5Co_{19}B_6$	125302 [46]
$NdFe_4B-\beta$	-0.081	0.081	65.19 (0.135)	0.241	$NdNi_4B$	2069928 [47]
$Nd_3Fe_{13}B_2$	-0.027	0.081	36.12 (0.144)	2.961	$Ce_3Ni_{13}B_2$	1778822 [48]
$Nd_3Fe_{11}B_4$	-0.131	0.085	28.22 (0.122)	0.150	$Ce_3Co_{11}B_4$	1852403 [49]
$Nd_2Fe_3B_6$	-0.375	0.088	16.02 (0.066)	0.132	$Ce_2Re_3B_6$	1966804 [50]
$NdFe_4B_4$	-0.342	0.090	17.30 (0.048)	0.140	$CeRu_4B_4$	2074891 [51]
$NdFe_2B_2-\beta$	-0.297	0.092	7.25 (0.057)	0.142	$CeIr_2B_2$	180315 [52]
$Nd_3Fe_8B_6$	-0.249	0.094	16.06 (0.079)	0.543	$Eu_3Rh_8B_6$	1771853 [53]
$Nd_2Fe_7B_3$	-0.147	0.096	35.04 (0.116)	0.209	$Ce_2Co_7B_3$	2016489 [54]

TABLE II. Ten-times ten-fold cross-validation results provided by the KRR model in predicting formation energy.

Model	R^2	MAE (eV/atom)	RMSE (eV/atom)
Kernel ridge	0.990(1)	0.094(2)	0.137(1)

TABLE III. Evaluation results of KRR, LG, DT models, and unsupervised GMM in estimating the stability of materials in $\mathcal{D}^{subst}_{\text{Nd-Fe-B}}$.

Model	*Precision*	*Recall*	f_1
KRR model	0.533	0.534	0.376
LG-model	0.629	0.687	0.599
DT-model	0.704	0.676	0.687
GMM	**0.729**	**0.821**	**0.735**

TABLE IV. Classification results in predicting "potentially formable" class label of substituted materials with KRR, LG, DT models, GMM, and ensemble models. The AND and OR operators in these ensemble models are denoted by "&" and "|", respectively

.

	KRR	LG	DT	GMM	KRR|GMM	LG|GMM	DT|GMM	KRR & GMM	LG & GMM	DT & GMM
Precision	0.24	0.35	0.56	0.49	0.24	0.36	0.48	**0.58**	0.53	**0.58**
Recall	0.82	0.79	0.45	0.91	0.97	**1.0**	0.91	0.76	0.7	0.45
f_1	0.37	0.49	0.5	0.64	0.39	0.53	0.63	**0.66**	0.61	0.51

TABLE V. Classification results in predicting "unstable" class label of substituted materials with KRR, LG, DT models, GMM, and ensemble models. The AND and OR operators in these ensemble models are denoted by "&" and "|", respectively.

	KRR	LG	DT	GMM	KRR|GMM	LG|GMM	DT|GMM	KRR & GMM	LG & GMM	DT & GMM
Precision	0.83	0.91	0.85	0.97	0.94	**1.0**	0.97	0.92	0.91	0.85
Recall	0.25	0.59	0.90	0.73	0.14	0.49	0.72	0.84	0.83	**0.91**
f_1	0.38	0.71	0.87	0.83	0.24	0.66	0.83	**0.88**	0.86	0.**88**

TABLE VI. Ternary phases of Nd-Fe-B compounds: formation energy E_f (eV/atom) and the stability calculated by DFT, ΔE^{DFT}, (eV/atom) given by OQMD.

Compound	E_f (eV/atom)	ΔE^{DFT} (eV/atom)	Stability state
$NdFe_4B_4$	-0.432	0.000	Potentially formable
$Nd_5Fe_2B_6$	-0.390	0.000	Potentially formable
$NdFe_{12}B_6$	-0.281	0.022	Potentially formable
$Nd_4Fe_3B_6$	-0.286	0.118	Unstable
Nd_2FeB	0.446	0.689	Unstable
$NdFe_2B$	0.775	1.018	Unstable
$NdFeB_2$	0.714	1.145	Unstable
